# Shielding working-memory representations from temporally predictable external interference

**DOI:** 10.1016/j.cognition.2021.104915

**Published:** 2021-12

**Authors:** Daniela Gresch, Sage E.P. Boettcher, Freek van Ede, Anna C. Nobre

**Affiliations:** a Department of Experimental Psychology, University of Oxford, Oxford, UK; b Oxford Centre for Human Brain Activity, Wellcome Centre for Integrative Neuroimaging, Department of Psychiatry, University of Oxford, Oxford, UK; c Institute for Brain and Behavior Amsterdam, Department of Experimental and Applied Psychology, Vrije Universiteit Amsterdam, The Netherlands

**Keywords:** Working memory, Temporal expectation, Distraction, Interruption, Attention

## Abstract

Protecting working-memory content from distracting external sensory inputs and intervening tasks is an ubiquitous demand in daily life. Here, we ask whether and how temporal expectations about external events can help mitigate effects of such interference during working-memory retention. We manipulated the temporal predictability of interfering items that occurred during the retention period of a visual working-memory task and report that temporal expectations reduce the detrimental influence of external interference on subsequent memory performance. Moreover, to determine if the protective effects of temporal expectations rely on distractor suppression or involve shielding of internal representations, we compared effects after irrelevant distractors that could be ignored vs. interrupters that required a response. Whereas distractor suppression may be sufficient to confer protection from predictable distractors, any benefits after interruption are likely to involve memory shielding. We found similar benefits of temporal expectations after both types of interference. We conclude that temporal expectations may play an important role in safeguarding behaviour based on working memory – acting through mechanisms that include the shielding of internal content from external interference.

## 1 Introduction

Visual working memory is the cognitive ability to store and manipulate visual information temporarily for guiding future behaviour (Baddeley, 1992; Nobre & Stokes, 2019). In everyday life, a key challenge for this memory system is to maintain task-relevant past sensations, while simultaneously ignoring external interference, such as irrelevant perceptual inputs (i.e., distractions) or intervening tasks (i.e., interruptions) (Bae & Luck, 2019; Berry, Zanto, Rutman, Clapp, & Gazzaley, 2009; Clapp, Rubens, & Gazzaley, 2010; Hakim, Feldmann-Wüstefeld, Awh, & Vogel, 2021; Mishra, Zanto, Nilakantan, & Gazzaley, 2013; Zickerick et al., 2020, Zickerick et al., 2021). Yet, factors contributing to the resilience of memory representations to sources of external interference remain poorly understood. Here, we investigated whether temporal expectations concerning interfering events could help mitigate their detrimental impact on working-memory performance. In addition, we asked if potential benefits of temporal expectations arise through improved suppression of anticipated external inputs or shielding (i.e., protection) of internal representations.

It has become well established that external distractors do not only interfere with the encoding (e.g., Feldmann-Wüstefeld & Vogel, 2019; McNab & Klingberg, 2008; Vogel, McCollough, & Machizawa, 2005; for a review see: Liesefeld, Liesefeld, Sauseng, Jacob, & Müller, 2020) but also with the retention of sensory information in working memory (for a review see: Lorenc, Mallett, & Lewis-Peacock, 2021). Accordingly, memory reports for both low-level features such as colour (Nemes, Parry, Whitaker, & McKeefry, 2012; Sun et al., 2017), location (Marini, Scott, Aron, & Ester, 2017; van der Stigchel, Merten, Meeter, & Theeuwes, 2007), orientation (Barth & Schneider, 2018; Lorenc, Sreenivasan, Nee, Vandenbroucke, & D'Esposito, 2018; Rademaker, Bloem, De Weerd, & Sack, 2015; Schneider, Barth, Getzmann, & Wascher, 2017), or motion (Berry et al., 2009; Pasternak & Zaksas, 2003), as well as high-level stimuli such as faces (Clapp et al., 2010; Mallett, Mummaneni, & Lewis-Peacock, 2020; Yoon, Curtis, & D'Esposito, 2006) become more prone to errors when a distractor is presented during the memory period. At the same time, working-memory content has also been reported to be quite robust to various forms of interference (Bettencourt & Xu, 2015; Rademaker, Chunharas, & Serences, 2019 [Experiment 1]; Zickerick et al., 2020; for a review see: Xu, 2017, Xu, 2020), alluding to dynamics that may support such resilience.

To understand how the impacts of interference on working memory can be reduced, we first turn to findings from the domain of perception. Studies on distraction during perceptual tasks have shown that learned regularities can help dampen the effects of distractors through proactive suppression (as reviewed in Geng, 2014; Geng, Won, & Carlisle, 2019; van Moorselaar & Slagter, 2020). For instance, salient distractors cause less interference when occurring at locations where they are more frequently presented (Failing, Feldmann-Wüstefeld, Wang, Olivers, & Theeuwes, 2019; Ferrante et al., 2018; Goschy, Bakos, Müller, & Zehetleitner, 2014; Huang, Vilotijević, Theeuwes, & Donk, 2021; Leber, Gwinn, Hong, & O'Toole, 2016; Reder, Weber, Shang, & Vanyukov, 2003; Sauter, Liesefeld, Zehetleitner, & Müller, 2018; Stilwell, Bahle, & Vecera, 2019; Wang & Theeuwes, 2018). Moreover, when targets appear at locations that frequently contain a salient distractor, detection of the target is slowed, suggesting a general suppression for the distractor location (Failing et al., 2019; Ferrante et al., 2018; Reder et al., 2003; Wang & Theeuwes, 2018). Extending these findings, efficient reduction of distractions can also rely on feature-based regularities. Salient distractors can be more efficiently rejected when they are more likely presented in one colour than any other colour (Failing et al., 2019; Feldmann-Wüstefeld & Schubö, 2016; Gaspelin & Luck, 2018; Stilwell et al., 2019; Vatterott & Vecera, 2012).

Similar to the perceptual domain, working memory may be more resilient to interference if distraction can be successfully predicted. There is some evidence that expectations about the occurrence of distractors can help counter their negative consequences during working memory. For example, working-memory performance has been shown to improve in conditions where distraction during retention was more likely and could therefore be anticipated, as opposed to those where distraction occurred only rarely (Hakim, Feldmann-Wüstefeld, Awh, & Vogel, 2020). However, it remains unclear whether other types of expectations can also mitigate distraction during working memory, or through what processes such mitigation might be mediated.

Temporal expectations of distractor onset could provide a potent source of information to guide proactive anticipation, thereby facilitating the handling of interference during working memory. In perception, besides spatial and feature information, the temporal dimension serves as a core facet of proactive anticipation, orienting attention selectively to relevant points in time (Coull & Nobre, 1998; van Ede, Rohenkohl, Gould, & Nobre, 2020; Vangkilde, Coull, & Bundesen, 2012; for a review see: Nobre & van Ede, 2018). Temporal expectations can improve performance when anticipated targets are followed by (van Ede, Chekroud, Stokes, & Nobre, 2018), paired with (Menceloglu, Grabowecky, & Suzuki, 2017), or embedded in distractors (Boettcher, Shalev, Wolfe, & Nobre, n.d.). Beyond biasing attention during perception, temporal expectations also operate in working memory, prioritising memory representations during times when they are anticipated to be most relevant for behaviour (Heuer & Rolfs, 2021; Jin, Nobre, & van Ede, 2020; Olmos-Solis et al., 2017; van Ede, Niklaus, & Nobre, 2017; Zokaei, Board, Manohar, & Nobre, 2019). To date, however, it has remained unaddressed whether temporal expectations can similarly help reduce potential sources of interference during working memory, and thereby facilitate working-memory performance.

At least two mechanisms could contribute to a potential benefit of temporal expectations in mitigating interference: First, sensory processing of task-irrelevant distractors may be suppressed (Bonnefond & Jensen, 2012; de Vries, Savran, van Driel, & Olivers, 2019; Getzmann, Wascher, & Schneider, 2018; Payne, Guillory, & Sekuler, 2013; Sawaki & Luck, 2011), without necessarily affecting internal representations. Alternatively (or additionally), anticipating interference may act on memory contents directly (i.e., shielding the memory items themselves) independently of affecting the processing of external interference per se.

To test for effects of memory shielding, it is necessary to introduce a type of interference that cannot simply be suppressed, such as a sensory input that requires a response (i.e., a secondary task) during the working-memory retention period. We therefore tested the putative benefits of temporal expectations when faced with two types of interference: to-be-ignored perceptual distractors and to-be-responded-to interrupters – the latter typically exerting a more detrimental influence over working memory (Bae & Luck, 2019; Berry et al., 2009; Clapp et al., 2010; Hakim et al., 2021; Mishra et al., 2013; Wang, Theeuwes, & Olivers, 2019; Zickerick et al., 2021). While suppression can be used to mitigate the effects of task-irrelevant perceptual distractors, it would be counterproductive to inhibit sensory information that is relevant for an intervening secondary task. Consequently, if we can demonstrate that proactive anticipation of a secondary task also improves memory performance, this would suggest a contribution from internal shielding beyond any potential influence from external distractor suppression.

## 2 Methods

### 2.1 Participants

The online study was approved by the Central University Research Ethics Committee of the University of Oxford.

Participants were recruited via Prolific Academic (https://www.prolific.co/) a platform for online participant recruitment well-suited for conducting web-based academic research (Palan & Schitter, 2018; Peer, Brandimarte, Samat, & Acquisti, 2017; Sauter, Draschkow, & Mack, 2020). Participants were pre-screened based on demographic criteria (i.e., age range 18 to 40, fluent in English), general health (i.e., normal or corrected-to-normal vision, no history of mental illnesses) and previous participation history on Prolific Academic (i.e., participated in at least 10 studies, with a study approval rate above 90%). All participants provided informed consent prior to participation and were paid £6.88 for their time. An additional monetary reward of up to £5 could be earned depending on participants' task performance in the experiment. Specifically, performance above 80% received a bonus payment scaling from £0.01 at 80% to £5 at 100%, with an average bonus payment of £0.76 (*SD* = 0.82) across all participants.

An initial power analysis in G*Power (Faul, Erdfelder, Lang, & Buchner, 2007) targeted on the detection of medium effects (*d* = 0.5, α = 0.05, 1-β = 0.95) suggested a sample size of *n* = 54. To reach the desired sample size, data were collected from 79 online participants. Data from 22 participants were excluded following our a-priori defined trial-removal procedure (before splitting data by conditions) and three more participants were removed because they reported utilising an explicit non-memory-based strategy to complete the task (see ‘Analysis’ for details). This yielded the desired final sample of 54 participants (age range: 18 to 38 years; mean age: 27.17 years; 28 females, 48 right-handed).

### 2.2 Task and procedure

**Fig. 1:**
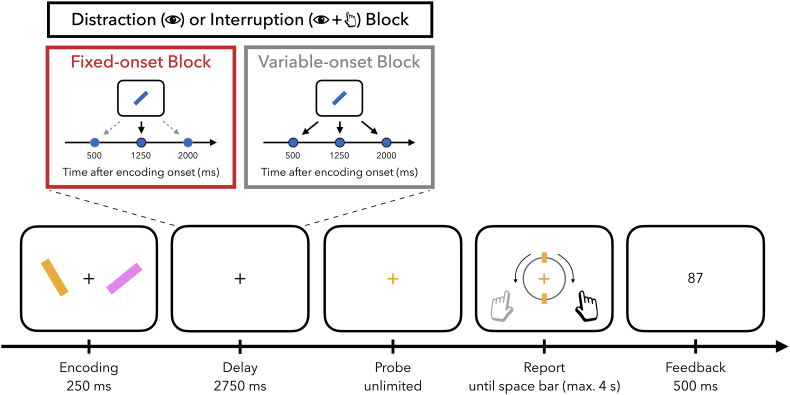
Task schematic. Trials started with an encoding display consisting of two, lateralised tilted bars. Participants' primary task was to remember the angle of both bars, of which one had to be reported at the end of the trial. On 75% of the trials within a block, interference occurred for 250 ms during the memory delay. In Distraction blocks, participants could ignore the interfering task-irrelevant distractor item. In Interruption blocks, participants indicated whether the interfering item was tilted to the left or to the right. In Fixed-onset blocks, interference occurred at a fixed time point within a block (at 500, 1250, or 2000 ms after encoding onset; counterbalanced across blocks). In Variable-onset blocks, interference occurred equally likely at each of the three possible time points. The delay between encoding offset and probe onset was always 2750 ms long. After the delay, a colour change of the central fixation cross indicated which bar's angle had to be reported. Participants were given unlimited time to retrieve the item from working memory and to decide what to report. However, once they started pressing a key, they were given only 4000 ms to complete their report. Following the report, participants received feedback in form of a number ranging from 0 to 100.

In the present study, participants performed a web-based visual working-memory task requiring the reproduction of the exact angle of one out of two tilted bars at the end of a memory delay (Fig. 1). Two main manipulations of this task were (1) that the interference either appeared at a fixed (i.e., predictable) or variable (i.e., unpredictable) point in time, and (2) that it was either an entirely irrelevant stimulus that should be ignored (i.e., distractor) or a stimulus requiring a response (i.e., interrupter). These manipulations allowed us to assess whether and how temporal expectations mitigate external interference in working memory. We return to these task features at the relevant instances below.

Participants completed the experiment in a web browser on their personal computers. The recommended internet browsers were Mozilla Firefox and Google Chrome; participating via mobile phone or tablet was not allowed. Prior to the experiment, participants' individual screen resolution was estimated by asking them to adjust an image of a credit card such that it matched the size of a physical credit card. In this manner, we could calculate the ratio between the card image width in pixels and the actual card width in centimetres to obtain a measure of pixel density (i.e., pixel per cm). Together with the instructed viewing distance of approximately 60 cm (i.e., one arm's length away from the monitor), this allowed us to present stimuli in degrees of visual angle, regardless of monitor size (Li, Joo, Yeatman, & Reinecke, 2020). The experimental script was generated in PsychoPy (Peirce et al., 2019) and hosted online using Pavlovia (http://www.pavlovia.org/). The experimental code can be found here, https://osf.io/2b68n/.

At the start of each trial, two tilted bars were simultaneously presented against a grey (RGB value: [128,128,128]) background for 250 ms. One bar was always positioned to the left and the other to the right of the central fixation cross. Independent of location, one of the bars was tilted to the left (anticlockwise) and the other to the right (clockwise). In order to avoid angles too close to vertical and horizontal meridians, the items' angles were randomly drawn in increments of 5° between 5° (−5°) and 85° (−85°). Across trials, a leftward or rightward oriented bar was equally likely to appear in the left (right) position. The stimuli subtended approximately 0.8° in width and 5.7° in length and were centred at a viewing distance of 5.7° visual angle from fixation. At encoding, both lateralised items were equally likely to be probed, rendering them equally relevant.

Visual encoding was followed by a memory delay of 2750 ms, in which the fixation cross remained on the screen. In 75% of trials within a block, a tilted bar (referred to as ‘interfering item’) was presented during the memory delay in the centre of the screen for 250 ms. The total length of the memory delay was 2750 ms, regardless of whether the interference appeared in that trial or not. Depending on the type of block, this interfering item was either presented at a variable or fixed time point (temporal predictability: fixed onset vs. variable onset), and participants either had to ignore or respond to it (interference type: distraction vs. interruption).

In fixed-onset blocks, the interfering item always appeared at a specific point in time during retention (interference onset: 500 ms or 1250 ms or 2000 ms after encoding onset depending on the block). In variable-onset blocks the interfering item was equally likely to occur at any of these three time points. That is, within the interference trials of a given variable-onset block, one third of the trials contained the interfering item at 500 ms, one third at 1250 ms and one third at 2000 ms after encoding onset.

In distraction blocks, participants were instructed to ignore the interfering item. In contrast, in interruption blocks, participants were required to respond to the item; if the bar was tilted to the left (right), participants pressed the F (J) key on the keyboard with their left (right) index finger as fast as possible. Within blocks, left- and rightward angles of the interfering items were counterbalanced. The interfering item was always presented in a different colour than the two memory items that preceded it. Colour of the memory items and the interfering item were always drawn from a set of three highly distinguishable colours (RGB values: blue [0,225,228], orange [254,163,0], pink [253,142,253]). The colours used for the memory items and the interfering item varied randomly across trials. The interfering item had the same size as the memory items and its angle was also randomly drawn in increments of 5° between 5° (−5°) and 85° (−85°).

Directly following the memory delay, the fixation cross changed colour (referred to as ‘probe’) to indicate for which memory item the tilt should be reported. Participants were never probed about the interfering item. Following the appearance of the probe, participants had unlimited time to decide on their response. To report a leftward (rightward) angle, participants were asked to press the F (J) key on the keyboard using their left (right) index finger. After response initiation, a visual response dial was displayed on the screen, always starting in vertical position. The response dial had the same diameter as the length of the bars (5.7° degrees) and always appeared surrounding the fixation.

The dial rotated leftwards when pressing F and rightwards when pressing J (either holding key down or pressing key repeatedly; always in increments of 5°). Critically, the dial could only be rotated in the direction that was initially indicated by the participant. For example, if a participant started pressing the F key after the probe, the dial would only move leftwards, and it would therefore not be possible to move the dial towards the right with the J key. Since the response dial always started in a vertical position and because it could not be rotated beyond ±90°, a leftward (rightward) oriented bar could only be correctly reported with a left (right) key. As a consequence, the hand required for responding was directly linked to the angle of the bar that was probed. This builds on previous tasks from our lab (Boettcher, Gresch, Nobre, & van Ede, 2021; van Ede, Chekroud, Stokes, & Nobre, 2019), though we note that the specifics of this response implementation were not critical to the current study. Once participants started rotating the dial, they were given only limited time (4000 ms) to complete the angle reproduction. This was intended to encourage participants to recall the exact orientation before moving the dial. When the dial aligned with the remembered tilt of the item, participants pressed the space bar to verify their response and continue with the task.

Next, participants received feedback in the form of a number ranging from 0 to 100, with 100 indicating a perfect report and 0 indicating that the adjusted orientation was perpendicular to the angle of the probed item. Feedback was presented for 500 ms. However, if time to adjust the angle ran out, the message ‘Too slow’ was presented instead for 750 ms. In interruption blocks this was followed by a second feedback message if participants responded with the wrong key (i.e., ‘Wrong key! Use the correct key to respond to the distractor!’) or did not respond at all to the interfering item (i.e., ‘Respond to the distractor!’). To incentivise fast responses to the interrupter, participants also received a feedback message when their reaction time (RT) to the interrupter was slower than 750 ms (i.e., ‘Too slow! Respond faster to the distractor!’). In distraction blocks, in which participants had to refrain from responding to the distractor, a feedback message was displayed if participants responded to the interfering item (i.e., ‘Don't respond to the distractor!’). The distractor- and interrupter-specific feedback message was combined with an image reminding participants to press F (J) when the interfering item was tilted to the left (right) or to withhold their response, respectively. Feedback was presented for a minimum of 750 ms and until the space key was pressed in order to encourage participants to read the feedback message before being able to continue with the next trial. Trials were separated by an inter-trial interval randomly drawn between 500 and 800 ms.

The experiment consisted of 384 trials divided across 12 blocks (each including 32 trials). The blocks were split according to the type of interfering event. Six blocks included interrupters, while the other six included distractors. These were further subdivided depending on the temporal predictability of the interfering event, which had a fixed onset in three blocks (one block each of 500 ms, 1250 ms, or 2000 ms), and a variable onset in the other three blocks (pseudo-randomised to occur equiprobably at 500 ms, 1250 ms, or 2000 ms). As such, the total number of trials where the interfering event would appear at any one delay interval after encoding onset (e.g., 500 ms) was equal between the corresponding fixed-onset block and across the three variable-onset blocks.

The order of blocks was pseudo-randomised with the two possible interference types nested within block pairs of the same temporal predictability. For example, a fixed-onset block with one type of interference (e.g., distractors) was always followed by another fixed-onset block with the other type of interference (e.g., interrupters). This would then be followed by a pair of variable-onset blocks (with the order of fixed-onset and variable-onset pairs being counterbalanced across participants). The order of the potential interference type was randomised within each temporal-predictability pair. The order of the fixed-onset blocks with interference at 500, 1250, or 2000 ms after encoding onset was randomised across participants.

The interference type (i.e., distractor vs. interrupter) was made explicit before the start of each block by presenting participants an image of the trial sequence and a verbal reminder to either ignore or respond to the interfering item. For the sake of simplicity, we referred to distractor blocks as ‘Ignore blocks’ and interrupter blocks as ‘React blocks’. Participants were informed that they would never have to report the angle of the interfering item. However, they were not informed about the temporal predictability (i.e., fixed vs. variable) or about the three possible interference onsets (i.e., 500 ms, 1250 ms, 2000 ms). In order to become familiarised with the procedure of the experiment, participants performed 16 practice trials of the interruption block and 16 trials of the distraction block, both with variable interference onset. At the end of the experiment, participants were redirected to the survey website Qualtrics (http://www.qualtrics.com/) where they were asked about comprehension of the instructions, potential strategy used to complete the task, and whether they thought their data should be analysed. The whole experiment lasted approximately 50 min.

### 2.3 Analysis

Data were analysed in R Studio (RStudio Team, 2019). During data pre-processing, trials were removed if RTs (i.e., from probe onset to the first key press) exceeded 5000 ms, were 2.5 SD above the individual mean across trials of all conditions, or if participants did not reproduce the probed angle within 4000 ms. We also excluded trials in distractor blocks if participants responded to the interfering item, as well as trials in interrupter blocks if participants did not respond, responded with the wrong key, or did not respond within 1000 ms to the interfering item. Twenty-two datasets where more than 10% of trials were rejected during these pre-processing steps were removed from further analysis. Additionally, three datasets were also removed where participants self-reported to have employed explicit non-memory-based strategies to maintain the encoding display (e.g., aligning their fingers with the memory items). After this exclusion step, the data of 54 participants with an average of 94.00% (*SD* = 1.82) retained trials entered the main analysis. Detailed information regarding the removal of trials per participant can be found in the analysis script.

Reproduction errors were calculated by averaging the absolute difference between the original angle of the target (i.e., probed) item and the reported angle across all trials and within each condition. We also examined RTs to the intervening task during interruption blocks. Moreover, we additionally, analysed the angular deviation between the angle of the interfering item and the reported angle.

To analyse the proportion of trials in which the observer incorrectly reported the angle of the interfering item (i.e., swaps, non-target responses), we fitted a mixture model to our data (Bays, Catalao, & Husain, 2009). The relative proportion of the non-target (i.e., interfering item) distribution was estimated separately for each participant and condition (i.e., interference type and temporal predictability). The mixture modelling analysis was performed using the ‘mixtur’ package (version 1.0.0; Grange & Moore, 2020).

As in previous studies (Lorenc et al., 2018; Mallett et al., 2020; Nemes et al., 2012; Rademaker et al., 2015; Sun et al., 2017; van der Stigchel et al., 2007), we also quantified whether the angle reported during recall of the memory item was biased either towards (i.e., attractive bias) or away from (i.e., repulsive bias) the angle of the interfering item. To this end, we first subtracted each participant's mean reproduction error calculated across all conditions from their reproduction error in each individual trial to eliminate general response biases. Next, the reproduction errors were sorted ordinally and then binned (i.e., moving-window approach, step size = 5°, bin width = 45°) according to the relative orientation of the target angle with respect to the interference angle. The average response bias was then calculated for each bin, yielding a response-bias curve as a function of the relative target-interference angular difference. This resulted in a response-bias value representing the degree to which the reported angle deviated from the target angle, where a negative value would indicate the report to be shifted clockwise, and a positive value would indicate the report to be shifted anticlockwise. Similarly, when the interference was oriented more clockwise or anticlockwise relative to the target, the target-interference angular difference would be negative or positive, respectively. As such, an attractive bias was indicated when the sign of the response bias matched the sign of the target-interference angular difference, whereas an opposing sign of the response bias relative to the relative target-interference angular difference indicated a repulsive bias. To test whether the interference biased memory reports, the area under the bias curve was integrated – separately for negative and positive target-to-interference angles – and subsequently compared for each participant across all conditions. As a last step, we examined whether temporal predictability or interference type might influence the magnitude of the response bias. Therefore, we equated the average response biases for the negative and positive interference-target angular differences of each participant and condition, then compared the resulting equated biases between factors.

When comparing more than two means, we applied a repeated-measures analysis of variance (ANOVA) and reported η^2^_G_ as a measure of effect size. We computed 2 × 2 repeated-measures ANOVA with the factors interference type (i.e., distraction vs. interruption) and interference presentation (i.e., no interference vs. interference) to test whether interference has a detrimental impact on working-memory performance. Moreover, we performed a 2 × 2 × 3 repeated-measures ANOVA with the factors temporal predictability (i.e., fixed vs. variable), interference type (i.e., distraction vs. interruption), and interference onset (i.e., 500 ms vs. 1250 ms vs. 2500 ms) to compare differences in reproduction errors. A 2 × 2 repeated-measures ANOVA with the factors temporal predictability and interference onset was performed to compare differences in RTs to the interrupter. Moreover, we computed a 2 × 2 repeated-measures ANOVA with the factors temporal predictability and interference type to test for differences in swaps in response biases. When evaluating only two means we applied paired samples *t*-test and report Cohen's *d* as a measure of effect size. For post hoc *t-*tests, we report Bonferroni-corrected *p* values that we denote as “*p*_Bonferroni_”. The ggplot2 package (version 3.3.3; Wickham, 2009) was used for plotting results. Where relevant, the within-subject standard error of the mean was calculated from normalised data using the approach from Morey (2008). The analysis script and data can be found here, https://osf.io/2b68n/.

## 3 Results

### 3.1 Interference has a detrimental impact on working-memory performance

**Fig. 2:**
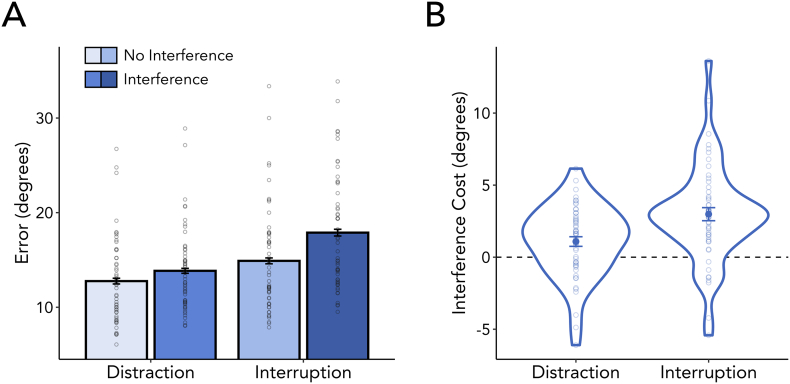
Both types of interference impair working memory. (A) Participants were less accurate in trials with as opposed to without interference. Each dot represents the mean reproduction error of one participant. (B) In both distraction and interruption blocks, working-memory performance decreased when interference was present. The interference cost of each interference type was calculated by taking the difference in reproduction errors between trials with and without interference (Interference – No Interference). Individual participants' differences are plotted as dots.

We first wanted to confirm the negative impact of both types of interference on the accuracy of reports associated with our working-memory task. To this end, we evaluated the average reproduction error (i.e., the absolute deviation from the probed orientation, for which lower levels indicate better performance) and compared trials with and without interference during the memory delay for distraction and interruption blocks. As depicted in Fig. 2A, errors were systematically larger when the memory delay was disrupted by interference in comparison to trials without interference (*F*_(1,53)_ = 53.868, *p* < 0.001, η^2^_G_ = 0.038). This was true for nearly every participant in our online experiment (Fig. 2B). We also found a significant effect of interference type (*F*_(1,53)_ = 73.667, *p* < 0.001, η^2^_G_ = 0.083), indicating that working-memory performance was worse in interruption as compared to distraction blocks. Further, although the interaction between interference presentation and interference type was significant (*F*_(1,53)_ = 11.044, *p* = 0.002, η^2^_G_ = 0.009), post-hoc pairwise comparisons revealed that in both distraction (*t*_(53)_ = −3.248, *p*_Bonferroni_ = 0.004, *d* = 0.442) and interruption blocks (*t*_(53)_ = −6.588, *p*_Bonferroni_ < 0.001, d = 0.897), working-memory performance was significantly decreased when interference was present.

### 3.2 Possible influence of temporal expectations on interference during working memory

**Fig. 3:**
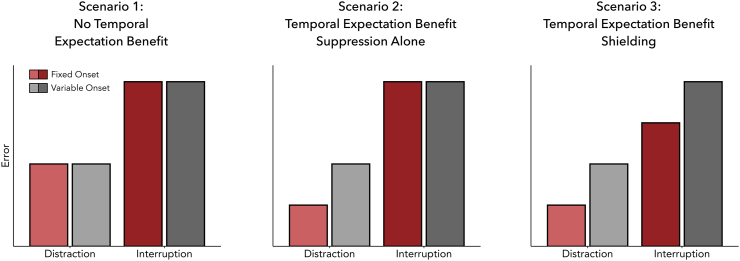
Possible patterns of errors in the working-memory task as a function of temporal predictability and interference type. No temporal expectation benefit (left), temporal expectation benefit occurs exclusively through distractor suppression (middle), and temporal expectation benefit occurs through memory shielding (right).

Having confirmed that interference negatively affected task performance, we next turned to our main question: Can proactive temporal expectations about the interference onset help overcome these detrimental effects? We considered three possible scenarios regarding how temporal expectations might influence working memory. First, temporal expectations may be unable to mitigate interference resulting in reproduction errors being similar between fixed and variable interference onsets (Fig. 3, Scenario 1, red vs. grey bars). Conversely, temporal expectations may help mitigate the effects of interference. Mitigation could occur through two mechanisms, reflected in two predicted patterns of results. If temporal expectations work only through suppressing external sources of interference, it should only reduce errors after distractors but be ineffective after interruption (Fig. 3, Scenario 2). Alternatively, if temporal expectations can shield directly internal representations from external sources of interference, errors should also be reduced for temporally predictable interrupters (Fig. 3, Scenario 3).

### 3.3 Temporal expectations mitigate interference

**Fig. 4:**
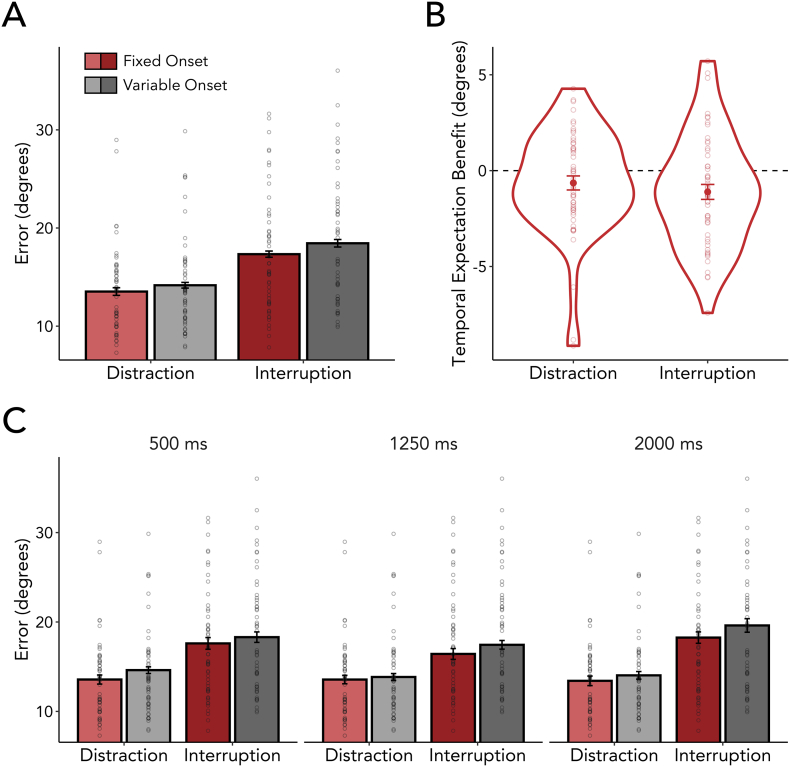
Temporal expectations about the onset of interfering events support the robustness of visual working memory. (A) Reproduction errors in the visual working-memory task were smaller when participants could temporally predict interference. Each dot represents the mean reproduction error of one participant. (B) Temporal expectations increased working-memory performance for both interference types. The temporal expectation benefit of each interference type was calculated by taking the difference in reproduction errors between variable-onset blocks and fixed-onset blocks (Fixed – Variable). Individual participants' differences are plotted as dots. (C) The temporal expectation effect was similar across all possible interference onsets. Each dot represents the mean reproduction error of one participant.

If participants could leverage temporal expectations to mitigate interference, then working-memory performance should be better (i.e., smaller reproduction errors) in blocks where interference occurred at a fixed (i.e., temporally predictable) as compared to a variable (i.e., temporally unpredictable) point in time during the memory delay. In support of this hypothesis, we found significantly smaller errors when interference could be temporally predicted (Fig. 4A; *F*_(1,53)_ = 8.774, *p* = 0.005, η^2^_G_ = 0.004), ruling out the first of our hypothetical scenarios (Fig. 3, left). In addition, and as expected, we also found a main effect of interference type, showing that participants were overall worse when they were required to respond to the interfering item (Fig. 4A; *F*_(1,53)_ = 75.289, *p* < 0.001, η^2^_G_ = 0.096).

### 3.4 Temporal expectations shield internal representations from anticipated interference

If participants benefited from temporal expectations exclusively by suppressing the interfering item – as in the second possible scenario (Fig. 3, middle) – then the effect of temporal expectations should be present only in distractor blocks, but not in interrupter blocks where participants had to attend and respond to the interfering item. Alternatively, if proactive anticipation in time instead (or additionally) shields working-memory content, then we should find smaller errors for fixed compared to variable onsets for both types of interference (Fig. 3, right). In line with the third scenario, the effects of temporal expectations were similar in distractor and interrupter blocks (Fig. 4A and B), without a significant interaction between temporal predictability and interference type (*F*_(1,53)_ = 0.502, *p* = 0.482, η^2^_G_ < 0.001). If anything, the temporal expectation benefit was numerically even larger, albeit not significantly, for interrupters than distractors. This suggests that temporal expectations help overcome interference not only when the source of interference can be ignored, but also when a secondary task must be completed during the period of memory retention.

This pattern of results was obtained across all three tested interference onsets (Fig. 4C), indicating that internal representations can be protected against distractors as well as interrupters, regardless of whether interference occurs at an early, intermediate, or late time point after encoding onset (see Supplementary Tables 1 for 2 full set of descriptive and inferential statistics).

### 3.5 The benefit on the working-memory task does not occur at the expense of the intervening task

**Fig. 5:**
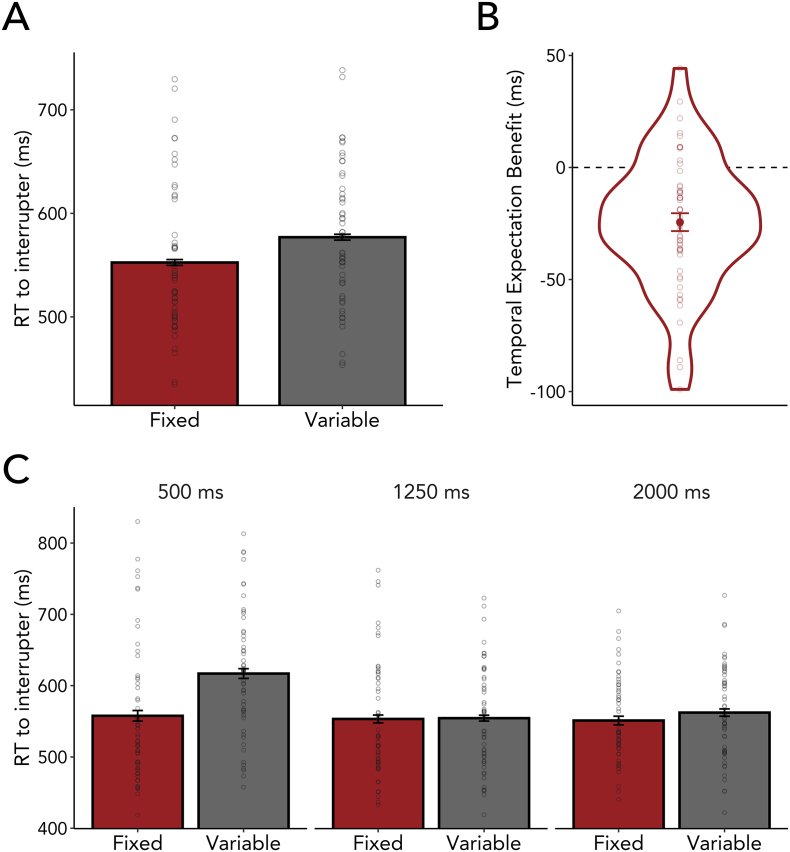
Temporal expectation benefit in the interruption (secondary) task. (A) Reaction times (RTs) to the interrupter were higher for fixed as compared to variable onsets. Each dot represents the mean reproduction error of one participant. (B) shows the difference in RTs to the interrupter between trials where the interrupter occurred at a fixed versus a variable point in time during memory retention (Fixed – Variable), with individual participants' differences plotted as dots. (C) The temporal expectation effect was most pronounced when the interrupter occurred at 500 ms as compared to at 1250 ms or 2000 ms after encoding onset. Each dot represents the mean reproduction error of one participant.

To rule out the possibility that participants experienced less interference simply because they chose to ignore the temporally predictable interrupters, we also tested for differences in RTs to the interrupters themselves, when these occurred at predictable vs. unpredictable times. Participants responded faster to the interrupter when it occurred at a predictable time in the memory delay compared to a variable onset (Fig. 5A and B; *F*_(1,53)_ = 32.037, *p* < 0.001, η^2^_G_ = 0.023). Thus, temporal expectations did not induce a trade-off between performance on the main and the intervening task, but instead improved performance on both.

For performance to the interrupter, we also found a main effect of interference onset (Fig. 5C; *F*_(2,106)_ = 15.655, *p* < 0.001, η^2^_G_ = 0.037). Overall, RTs occurred faster when the interrupter was presented at 1250 ms (*t*_(53)_ = 5.123, *p*_Bonferroni_ < 0.001, *d* = 0.697) and 2000 ms (*t*_(53)_ = 4.089, *p*_Bonferroni_ < 0.001, *d* = 0.556) as compared to 500 ms; however, there was no difference in RTs between trials with 1250-ms and 2000-ms interrupter onsets (*t*_(53)_ = −0.368, *p*_Bonferroni_ = 1.000, *d* = 0.050).

Interestingly, in contrast to what we observed for the influence of temporal expectations on working-memory performance (Fig. 4C), we found a significant interaction between temporal predictability and interference onset in the secondary task (Fig. 5C; *F*_(2,106)_ = 15.785, *p* < 0.001, η^2^_G_ = 0.026). Pairwise comparisons revealed faster RTs to the interrupter when it was temporally predictable compared to unpredictable at 500 ms (*t*_(53)_ = 6.065, *p*_Bonferroni_ < 0.001, *d* = 0.825), but not at 1250 ms (*t*_(53)_ = 0.187, *p*_Bonferroni_ = 1.000, *d* = 0.025) or 2000 ms (*t*_(53)_ = 1.709, *p*_Bonferroni_ = 0.280, *d* = 0.233). The effect of faster RTs to interrupters after short delays but not after long delays is in agreement with prior studies of temporal expectations in simple perception and action tasks (Coull, Frith, Büchel, & Nobre, 2000; Cravo, Rohenkohl, Santos, & Nobre, 2017; Miniussi, Wilding, Coull, & Nobre, 1999); Nobre, 2001; also reviewed in Nobre, 2001; Nobre & van Ede, 2018). Interestingly, however, we did not find a similar onset dependence for the protective effect of temporal expectations on working-memory performance (Fig. 4C). This may suggest that temporal expectations exert distinct influences on working-memory protection and secondary task facilitation – a possibility that remains interesting to address in future research but for which further discussion is beyond our current scope.

### 3.6 Temporal expectations shield internal representations instead of averting external interference

**Fig. 6:**
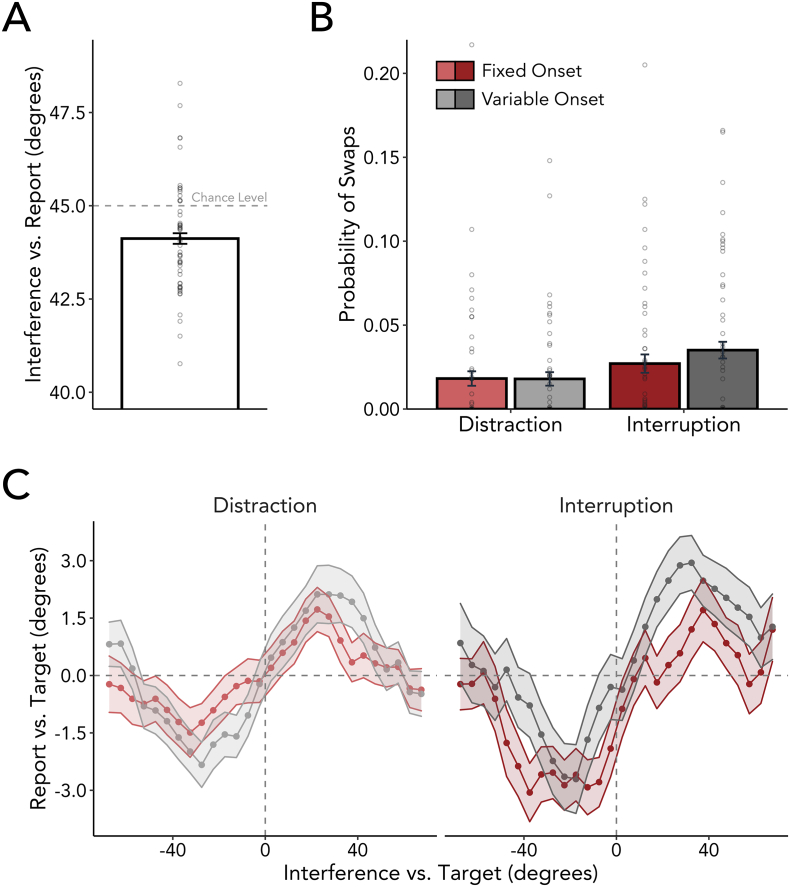
Encoding of interference into working memory. A) There is a significant difference between the angular deviation between interference and report and the chance level of 45° (depicted as dashed line). Each dot represents the mean reproduction error of one participant. (B) Interrupters as compared to distractors increased the swap rate, however temporal expectations did not affect the probability of reporting the interfering item. Each dot represents the mean reproduction error of one participant. (C) shows the average response bias relative to the angular difference between target and interfering item. A negative value along the y-axis would indicate reports biased clockwise from the angle of the target item, while a negative value along the x-axis would indicate that the interfering item's angle was clockwise from the target item's orientation. The memory reports were therefore shown to be biased towards the interference (e.g., an attractive bias). However, neither interference type nor temporal predictability modulated this attraction bias.

Although we demonstrated that temporal expectations help mitigate the detrimental effects of interference on working memory even when interfering events cannot be suppressed, it is not immediately possible to conclude unequivocally that shielding alone can account for the effects. An alternative possibility is that, while allowing for sensorimotor processing, temporal expectations may have modulated the degree to which interfering items became encoded into working memory. Such a mechanism would mitigate the impact of external information instead of targeted protection of internal representations. To consider this option, we firstly tested whether the interference angle entered working memory at all. Although participants were explicitly informed that they will never be probed about the angle of the interfering item, it might be possible that this information was still sometimes incidentally encoded. Using a pairwise comparison, we tested whether the angular deviation between interference and report differed from a chance level of 45 degrees. As depicted in Fig. 6A, the actual angular deviation between interference and report (*mean* = 44.121, *SD* = 1.045) significantly differed from chance (*t*_(53)_ = 4.372, *p* < 0.001, *d* = 0.595), suggesting that some information regarding the interfering item was carried over into the working-memory reporting stage.

We considered two possible explanations for the above chance reporting of the interfering item. First, on a subset of trials participants may be erroneously reporting the orientation of the interfering item rather than reporting the memorized item orientation, here termed a ‘swap’. Second, participants may be generally biased (or pulled) towards the orientation of the interfering item in their report of the memory item. If these effects are modulated by temporal expectations, then we can assume that the temporal expectation benefit is due at least in part to diminished encoding of the interfering item.

Using a mixture model approach according to Bays, Catalao, & Husain, 2009, we quantified the proportion of swaps with the interfering item as a function of temporal predictability and interference type (Fig. 6B). Although overall there was a low proportion of swap errors (< 5%), interrupters as opposed to distractors did significantly increase the probability of swaps (*F*_(1,53)_ = 6.512, *p* = 0.014, η^2^_G_ = 0.026). Crucially, however, temporal predictability did not affect the swap rate (*F*_(1,53)_ = 0.761, *p* = 0.387, η^2^_G_ = 0.002), and the interaction between the two factors did not reach significance (*F*_(1,53)_ = 0.811, *p* = 0.372, η^2^_G_ = 0.003).

These findings are complemented by the results of the response-bias analysis (Fig. 6C). Generally, the reproduction of the target angle was biased towards the interference angle (*t*(53) = 3.779, *p* < 0.001, *d* = 0.514). That is, when the interference orientation was anticlockwise (clockwise) to the target orientation there was an anticlockwise (clockwise) shift in participants' orientation reports of the target. Comparing this pull effect between conditions revealed no systematic difference between interrupters and distractors (*F*(1,53) = 1.308, *p* = 0.258, η^2^_G_ = 0.007) or between fixed and variable onset (*F*(1,53) = 0.707, *p* = 0.404, η^2^_G_ = 0.003). Moreover, there was no significant interaction between interference type and temporal predictability (*F*(1,53) = 0.117, *p* = 0.734, η^2^_G_ < 0.001).

Thus, even though interference entered working memory, being able to anticipate the timing of an interfering event did not lead to a difference in the memory trace of the distracting or interrupting event. As such, temporal expectations did not modulate the degree to which external information enters working memory. Instead, it is more plausible that temporal expectations affected how well internal representations are shielded against external disturbances.

## 4 Discussion

To ensure efficient goal-directed behaviour, internal representations must be protected from irrelevant perceptual distractors as well as intervening tasks. Here, we provide evidence that temporal expectations help overcome the detrimental impact of both types of interference on visual working memory. Our results demonstrate that temporal expectations improved working-memory performance irrespective of the type of interference – that is, even when interference acts as a secondary task. Because temporal expectations also improved working-memory performance in interrupter trials, we can conclude this benefit is unlikely driven solely by increased suppression of the external sensations. Instead, our findings suggest that shielding of internal contents may provide a potent source to mitigate interference during working-memory retention. This is further supported by the finding that temporal expectations protected working memory even when enhancing performance to the secondary task.

In addition to these main insights, we also replicate previous research demonstrating working memory to be substantially more impaired following interruptions as opposed to distractions (Bae & Luck, 2019; Berry et al., 2009; Clapp et al., 2010; Hakim et. al., 2021; Mishra et al., 2013; Wang et al., 2019; Zickerick et al., 2021). Besides calling on attentional control processes and mental workspaces that may have been concurrently active for working memory (e.g., Bae & Luck, 2019; Barrouillet, Bernardin, Portrat, Vergauwe, & Camos, 2007; Souza & Oberauer, 2017), interrupters – which required a manual response – may also have directly affected the preparation of memory-guided actions. In our task, items were associated with specific actions which would allow action plans to be coactivated together with visual representations (Boettcher et al., 2021; van Ede et al., 2019). As such, interrupters may have additionally interfered with action plans in working memory, yielding a more detrimental effect on performance than was elicited by visual distractors not requiring any manual response. In agreement with this, a recent study (Zickerick et al., 2021) showed that interrupters, but not distractors, were detrimental to the modulation of electroencephalography (EEG) mu-alpha activity – a neural signature linked to action preparation (McFarland, Miner, Vaughan, & Wolpaw, 2000; Neuper, Wörtz, & Pfurtscheller, 2006; Salmelin & Hari, 1994). Thus, in addition to the requirement to reactivate sensory representations after interruption (Clapp et al., 2010; Sakai, Rowe, & Passingham, 2002), the impeded retrieval of action plans following interrupters may further account for the greater memory loss – a possibility to be more thoroughly tested in future research.

Besides interrupters having a detrimental influence on working memory, perceptual distractors also impaired memory-guided performance relative to no interference. Interestingly, previous research has yielded mixed evidence regarding the ability of perceptual distractors to impair working-memory performance (Bettencourt & Xu, 2015; Rademaker et al., 2019 [experiment 1]; Zickerick et al., 2020; for a review see: Xu, 2017, Xu, 2020). However, in studies showing working-memory detriments, high categorical overlap between memory target and distractor is suggested to play an important role (for a review see: Lorenc et al., 2021). In the present work, the interfering item was highly similar to the memory content. Future studies will be informative to characterise more comprehensively how sensory and task-demand variables affect internal representations stored in working memory.

It has previously been shown that working-memory performance can improve when interfering items gained less attention during retention (Bonnefond & Jensen, 2012; Clapp et al., 2010). Building on this, we demonstrate that working memory can also improve through anticipation of interference, even when the source of the interference itself cannot be suppressed, as was the case for our interrupters. In fact, RTs were even faster for predictable early interrupters (i.e., 500 ms after encoding onset), indicating that increased attention to the interfering item can co-occur with better working-memory performance. This might potentially be mediated by active allocation of attention to expected interference, as recently demonstrated in a related working-memory task (Makovski, 2019). Thus, our results argue for a second route by which distractor anticipation can facilitate working-memory performance – by shielding of the internal representations, rather than suppressing the external inputs. Critically, we do not intend to suggest that shielding abolishes the detrimental effects of interfering events altogether. Instead, as we have demonstrated, working memory performance is better protected from sources of interference when these sources come at expected moments in time. Hence, while temporal expectations versus temporal uncertainty may not follow an all-or-none shielding rule, the key finding is that the ability to predict interference in time diminishes its impact on visual working memory.

Although our findings advocate for memory shielding, we do not wish to claim that distraction suppression is not also an important mechanism for handling interference. Previous work has demonstrated that perceptual distractors are suppressed while ongoing memory content is maintained (Bonnefond & Jensen, 2012; de Vries et al., 2019; Getzmann et al., 2018; Payne et al., 2013; Sawaki & Luck, 2011), more specifically, Bonnefond and Jensen (2012) found evidence for suppression through phase shifts within the alpha band prior to the onset of a temporally predictable distractor. Moreover, task-irrelevant distractors – but not interrupters which required attention – elicited neural signatures reflecting suppression (Hakim et al., 2021).

**Fig. 7:**
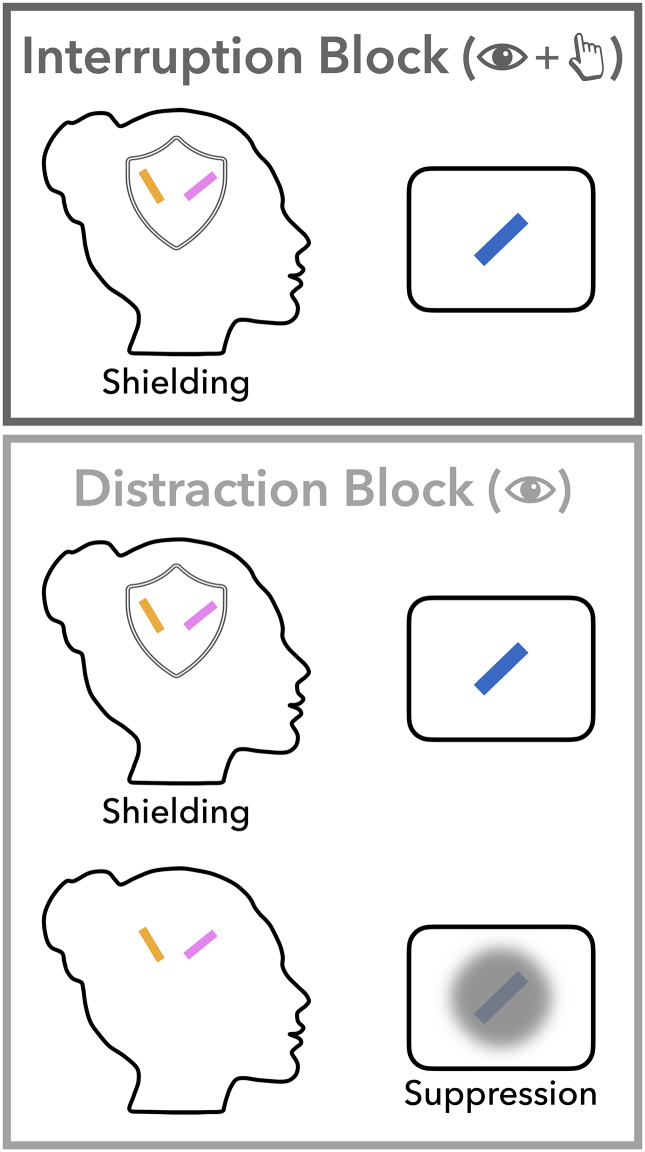
Shielding and suppression might operate independently based on current task demands. Our findings show that suppression alone cannot account for all our findings, as indicated by the temporal expectation benefit in interruption blocks. However, future studies need to determine whether perceptual distractors can only be suppressed, or whether working-memory representations can also be shielded against this type of interference.

Nonetheless, suppression cannot account for all our findings. If the observed effects were purely driven by suppression, working-memory performance would not improve when interference imposed secondary task demands requiring attention, and task performance to the interrupter would be similarly unlikely to improve. We showed that shielding (and not suppression) operates in interruption blocks, where suppression is not a viable option (Fig. 7, top). However, based on our present experimental design, we cannot determine whether suppression or shielding, alone or together, operated in distraction blocks (Fig. 7, bottom). Potentially, the anticipation of interference in our study may have been subserved both by shielding and suppression mechanisms acting independently and differentially based on task demands. Thus, in future work, it will be of interest to use neural measures to adjudicate between the two mechanisms. For instance, multivariate decoding of EEG signals with high temporal resolution may expose differential neural signatures linked to the handling of each interference type (c.f., van Ede et al., 2018). This could inform us whether there is a default mechanism by which temporal expectations are enacted to protect working memory, or whether the mechanism utilised depends on the task and source of interference at hand.

Furthermore, our study raises an interesting question regarding how the two mechanisms – suppression of external input and shielding of internal representations – could operate. Suppression of anticipated interference during working-memory retention has been linked to modulations of oscillatory activity, such as increases in midfrontal theta (de Vries et al., 2019) and posterior alpha power (Bonnefond & Jensen, 2012; de Vries et al., 2019; Payne et al., 2013), presumably reflecting an inhibition of sensory areas involved in processing of distractors. However, there is less consensus as to how memory contents themselves can be protected against interference. Over the past decade, different types of evidence have emerged regarding the neural mechanisms of how items are held in working memory (Kamiński & Rutishauser, 2020; Serences, 2016), each offering unique possibilities as to how internal representations could be shielded. For example, the traditional assumption that working memory relies on sustained neural firing (Funahashi, Bruce, & Goldman-Rakic, 1989; Fuster & Alexander, 1971; Goldman-Rakic, 1995; Miller, Erickson, & Desimone, 1996) has recently been challenged. ‘Activity-silent’ mechanisms have been proposed to underlie working-memory retention through synaptic weight changes (Masse, Rosen, & Freedman, 2020; Mongillo, Barak, & Tsodyks, 2008; Stokes, 2015). Based on the results of neural decoding analyses, it has been suggested that memory content may transition to a ‘latent’ state while other distractors or working-memory items are processed before re-emerging into active state when task relevant (LaRocque, Riggall, Emrich, & Postle, 2017; Lewis-Peacock, Drysdale, Oberauer, & Postle, 2012; Sprague, Ester, & Serences, 2016). Further, as these silent memories do not seem to bias perception (Mallett & Lewis-Peacock, 2018) or be manipulated without prior reactivation (Trübutschek, Marti, Ueberschär, & Dehaene, 2019), it is conceivable that information stored in a latent state might be less susceptible to disruption by interference (cf. Lorenc et al., 2021) – an exciting possibility that remains to be carefully investigated.

In the current work, the time of the working-memory task itself was always fully predictable. Because of this, the momentary task relevance of the memory contents could be deduced by the passage of time (as in Jin et al., 2020; Olmos-Solis et al., 2017; van Ede et al., 2017; Zokaei et al., 2019). Foreknowledge of when the memory contents become relevant may well play a role in the ability to shield internal representations from external interference, and it may allow internal representations to be momentarily deprioritised and facilitate interference handling. For example, temporal expectations regarding the onset of interference may serve to indicate when representations should be transformed into a format that is more resistant to interference, such as an orthogonal activity-silent code or a higher-order code in non-sensory areas. However, it should be noted that this assertion remains purely speculative and remains to be followed-up with dedicated neuroscientific investigations.

As a final point, we consider possible competing interpretations that could account for the shielding effect. First, it is possible that temporal expectations modulate the extent to which interference is encoded into working memory by altering gating mechanisms. When interference is temporally unpredictable, the likelihood that the interfering item will mistakenly enter working memory could be increased, thereby potentially displacing one of the relevant memory representations. Although our analyses indicated that interference is encoded to some extent or in some trials, there was no difference between temporally predictable and temporally unpredictable interference in the probability of swaps or the response bias, rendering this explanation unlikely. However, some caution may be pertinent when interpreting these null results. Alternatively, working memory might have been conserved by saving attentional resources when the interrupter task was temporally predictable. Yet, when splitting the data into the three interference onsets, we only found a significant effect of temporal expectation for interrupter RTs at 500 ms after encoding onset. In contrast, we demonstrate a temporal expectation benefit in working-memory performance following interrupters occurring at 500 ms, 1250 ms, and 2500 ms after encoding onset. The fact that we observe an effect of temporal expectations on working memory without effects on the interrupter task (i.e., for 1250 ms and 2500 ms) argues against the possibility that temporally predictable interrupters were simply less interfering because they saved attentional resources by enabling faster responses to the interfering item. Lastly, the necessity to divide and/or switch attention between internal (i.e., memory content) and external (i.e., interference) space might be reduced based on the temporal predictability of interference. Previous research demonstrated that the costs of switching between external and internal attention are similar in magnitude as within-domain switch costs (Verschooren, Schindler, De Raedt, & Pourtois, 2019). Being able to anticipate when interference will occur might lower the need to divide/switch attention between the two domains. Nevertheless, this alternative interpretation of our results does not discount the idea of memory shielding – with reduced demands to beware of the external event, internal attention can stay focussed on memory contents, thereby protecting them from anticipated interference.

In conclusion, the present study shows firstly that temporal expectations help mitigate interference during visual working memory, and additionally that the influence of proactive temporal anticipation of interference engages processes of memory shielding. In future studies, it will be interesting to reveal the exact (neural) mechanisms that support the handling of these various sources of interference in working memory.

## Author contributions

D.G., S.E.P.B., F.v.E., and A.C.N. designed the research; D.G. programmed experiment and performed data collection; D.G. and S.E.P.B. performed the main analyses and made the figures; D.G., S.E.P.B., F.v.E., and A.C.N. wrote and revised the manuscript.

## Appendix A Supplementary data

Supplementary material Image 1
