# Supplementary material for: Shielding working-memory representations from temporally predictable external interference
Source: Cognition. 2021 Dec;217:104915. doi: 10.1016/j.cognition.2021.104915 (PMC8543071; doi:10.1016/j.cognition.2021.104915)
Supplement: Supplementary file 1 — Supplementary material [file mmc1.pdf]

Supplementary material

Supplementary Table 1. Descriptive statistics. Mean and standard error of reproduction errors (SE) for the factors interference type (distraction vs. interruption), temporal predictability (fixed vs. variable), and interference onset (500 ms vs. 1250 ms vs. 2000 ms).

	Distraction						Interruption					
	Fixed			Variable			Fixed			Variable		
mean	13.526			14.167			17.334			18.444		
SE	0.390			0.289			0.317			0.383		
	500 ms	1250 ms	2000 ms	500 ms	1250 ms	2000 ms	500 ms	1250 ms	2000 ms	500 ms	1250 ms	2000 ms
mean	13.565	13.561	13.418	14.616	13.848	14.031	17.605	16.428	18.256	18.304	17.447	19.614
SE	0.507	0.471	0.535	0.375	0.388	0.429	0.656	0.607	0.637	0.586	0.485	0.758

Supplementary Table 2. (A) Main effects and interactions of reproduction errors for the factors temporal predictability (fixed vs. variable), interference type (distraction vs. interruption), and interference onset (500 ms vs. 1250 ms vs. 2500 ms), tested with a 2×2×3 repeated-measures ANOVA. This analysis yielded a significant main effect of temporal predictability, confirming the effect that temporal expectations improve visual working-memory performance. We also found a significant effect of interference type and interference onset. Further, the interaction between interference onset and interference type was significant. (B) Bonferroni-corrected pairwise comparisons of reproduction errors between the three different interference onsets were conducted to break down the main effect of the factor interference onset. However, errors did not significantly differ between the three interference onsets. (C) To test whether the effect of interference type differed between interference onsets, we performed Bonferroni-corrected pairwise comparisons. This analysis revealed that interrupters as compared to distractors significantly decreased visual working-memory performance at all three interference onsets. * indicates $p < 0.05$, ** indicates $p < 0.001$.

A) Repeated-measures ANOVA of reproduction errors

	df	*F*	*p*	η^2_G
Temporal Predictability*	1, 53	8.774	0.005	0.004
Interference Type**	1, 53	75.289	< 0.001	0.096
Interference Onset*	2, 106	3.395	0.037	0.004
Temporal Predictability × Interference Type	1, 53	0.502	0.482	< 0.001
Temporal Predictability × Interference Onset	2, 106	0.104	0.902	< 0.001
Interference Type × Interference Onset*	2, 106	3.943	0.022	0.004
Temporal Predictability × Interference Type × Interference Onset	2, 106	0.415	0.662	< 0.001

B) Bonferroni-corrected pairwise comparisons of reproduction errors between the three different interference onsets.

	df	*t*	$p_{Bonferroni}$	*d*
500 ms vs. 1250 ms	53	1.936	0.175	0.263
500 ms vs. 2000 ms	53	-0.722	1.000	0.098
1250 ms vs. 2000 ms	53	-2.192	0.098	0.298

C) Bonferroni-corrected pairwise comparisons of reproduction errors between distractors and interrupters at each of three interference onsets.

	df	*t*	$p_{Bonferroni}$	*d*
Distractor vs. Interrupter: 500 ms**	53	-6.101	< 0.001	0.830
Distractor vs. Interrupter: 1250 ms**	53	-5.572	< 0.001	0.758
Distractor vs. Interrupter: 2000 ms**	53	-7.839	< 0.001	1.067
